# Investigation of Delafloxacin Resistance in Multidrug-Resistant *Escherichia coli* Strains and the Detection of *E. coli* ST43 International High-Risk Clone

**DOI:** 10.3390/microorganisms11061602

**Published:** 2023-06-16

**Authors:** Dániel Gulyás, Katalin Kamotsay, Dóra Szabó, Béla Kocsis

**Affiliations:** 1 Institute of Medical Microbiology, Semmelweis University, 1089 Budapest, Hungary; gulyas.daniel@med.semmelweis-univ.hu (D.G.); szabo.dora@med.semmelweis-univ.hu (D.S.); 2 Central Microbiology Laboratory, National Institute of Hematology and Infectious Disease, Central Hospital of Southern-Pest, 1097 Budapest, Hungary; kamotsaykati@gmail.com; 3 Human Microbiota Study Group, Semmelweis University-Eötvös Lóránd Research Network, 1089 Budapest, Hungary

**Keywords:** fluoroquinolones, delafloxacin, multidrug-resistance, extended-spectrum beta-lactamase, *E. coli*

## Abstract

Delafloxacin is a novel fluoroquinolone agent that is approved for clinical application. In this study, we analyzed the antibacterial efficacy of delafloxacin in a collection of 47 *Escherichia coli* strains. Antimicrobial susceptibility testing was performed by the broth microdilution method and minimum inhibitory concentration (MIC) values were determined for delafloxacin, ciprofloxacin, levofloxacin, moxifloxacin, ceftazidime, cefotaxime, and imipenem. Two multidrug-resistant *E. coli* strains, which exhibited delafloxacin and ciprofloxacin resistance as well as extended-spectrum beta-lactamase (ESBL) phenotype, were selected for whole-genome sequencing (WGS). In our study, delafloxacin and ciprofloxacin resistance rates were 47% (22/47) and 51% (24/47), respectively. In the strain collection, 46 *E. coli* were associated with ESBL production. The MIC50 value for delafloxacin was 0.125 mg/L, while all other fluoroquinolones had an MIC50 value of 0.25 mg/L in our collection. Delafloxacin susceptibility was detected in 20 ESBL positive and ciprofloxacin resistant *E. coli* strains; by contrast, *E. coli* strains that exhibited a ciprofloxacin MIC value above 1 mg/L were delafloxacin-resistant. WGS analysis on the two selected *E. coli* strains (920/1 and 951/2) demonstrated that delafloxacin resistance is mediated by multiple chromosomal mutations, namely, five mutations in *E. coli* 920/1 (*gyrA* S83L, D87N, *parC* S80I, E84V, and *parE* I529L) and four mutations in *E. coli* 951/2 (*gyrA* S83L, D87N, *parC* S80I, and E84V). Both strains carried an ESBL gene, *bla*_CTX-M-1_ in *E. coli* 920/1 and *bla*_CTX-M-15_ in *E. coli* 951/2. Based on multilocus sequence typing, both strains belong to the *E. coli* sequence type 43 (ST43). In this paper, we report a remarkable high rate (47%) of delafloxacin resistance among multidrug-resistant *E. coli* as well as the *E. coli* ST43 international high-risk clone in Hungary.

## 1. Introduction

The spread of multidrug-resistant bacteria is a considerable challenge worldwide. In the wake of COVID-19, the circulation of antibiotic-resistant bacteria was enhanced, turning into a pandemic that threatens public health globally [1,2,3]. Antibiotic-resistant bacteria are the leading causative agents of severe infections, including bacteriaemia, pneumonia, and urinary tract infection. Usually, antibiotic-resistant bacterial infections occur in hospitalized patients, and these infections are associated with higher mortality rates. A recent study described that antibiotic-resistant bacterial infections correspond to 1.27 million patients’ deaths in a single year worldwide [4,5,6]. The major pathogens of antibiotic-resistant bacteria belong to the ESKAPE group, (*Enterococcus* spp., *Staphylococcus aureus*, *Klebsiella pneumoniae*, *Acinetobacter baumannii*, *Pseudomonas aeruginosa*, and *Enterobacter* spp.), which are responsible for the high number of difficult-to-treat infections, because a limited number of effective antibiotics are available for antibacterial therapy [7,8]. World Health Organization (WHO) released a priority list of antibiotic-resistant bacteria, where discovery and research are needed for effective antibiotics. On the list of the “Priority 1: critical” group, certain bacteria are present, namely, *A. baumannii*, *P. aeruginosa*, and Enterobacteriaceae, which are resistant to carbapenems and to third-generation cephalosporins [9]. 

In recent years, several novel antimicrobial agents were approved for clinical use that are effective against bacteria that are already resistant to commonly used antibiotics. Among these novel agents, we can find plazomicin, cefiderocol as well as beta-lactam and beta-lactamase-inhibitor combinations [10,11,12,13]. 

New fluoroquinolone agents, such as delafloxacin, finafloxacin, and zabofloxacin, with enhanced antibacterial efficacy were also approved for clinical application and were marketed in several countries [14,15,16,17]. Fluoroquinolones are nucleic-acid-synthesis-inhibitor antibacterial agents, targeting both bacterial gyrase and topoisomerase IV enzymes, which makes them suitable as products with broad-spectrum antibacterial efficacy [18,19,20,21]. Due to widespread fluoroquinolone resistance, the clinical indication of earlier fluoroquinolone agents (e.g., ciprofloxacin and levofloxacin) has been restricted [22,23]. 

Delafloxacin (whose earlier names were ABT-492, RX-3341, and WQ-3034) is a new fluoroquinolone agent with an anionic, non-zwitterionic structure. Delafloxacin demonstrates a potent bactericidal effect against both Gram-positive and Gram-negative bacteria because it inhibits both bacterial DNA gyrase and topoisomerase IV enzymes. The chemical structure of delafloxacin is 1-(6-amino-3,5-difluoro-2-pyridinyl)-8-chloro-6-fluoro-7-(3-hydroxy-1-azetidinyl)-4-oxo-1,4-dihydro-3-quinolinecarboxylate [14,18]. Based on its chemical structure, it is a weak acid, and it remains uncharged in acidic environment, which enables its transmembrane transfer into the bacterial cell, where it accumulates. In a neutral pH (e.g., in intracellular space), it stays in its anionic, deprotonated form and maintains its concentration-dependent antibacterial activity. The anionic feature of delafloxacin makes it suitable as a product with an enhanced antibacterial efficacy in acidic environments, for example, in inflammations of human tissues, in phagolysosomes, in infections of skin and soft tissue, and in abscesses [18,23]. Furthermore, delafloxacin has other features in its chemical structure. A heteroaromatic substitution is located on delafloxacin, which provides a larger molecular surface that enhances antibacterial activity against strains that are resistant to earlier fluoroquinolones, because delafloxacin can bind to more sites on the target molecules. A chlorine atom is also located on delafloxacin, which provides a strong polarity and enhances its efficacy against anaerobic bacteria [14,18]. Delafloxacin can be administered through the oral or intravenous way. In an oral administration of delafloxacin, a 450 mg dose is necessary to achieve a corresponding concentration–time profile, compared to that of the intravenous 300 mg dose. In certain cases, 58.8% of the oral bioavailability of delafloxacin can be decreased through pharmacological interactions (e.g., by chelation effects) with other medications that contain multivalent metal cations, such as Al^3+^, Mg^2+^, Fe^2+^, or Zn^2+^ [14,18,22,23]. Delafloxacin does not inhibit cytochrome P450 isoenzymes; therefore, it does not have clinically relevant drug–drug interactions with the most frequently used medicines. Interestingly, no potential synergistic or antagonistic effects were detected between delafloxacin and other commonly used antibiotics [14,18]. Approximately 84% of delafloxacin binds to plasma proteins. A higher dose of delafloxacin was administered to patients with kidney failure, but hepatic impairment did not significantly affect the antibacterial activity of delafloxacin [14]. The side effects of earlier fluoroquinolone agents are well known. Among the most frequent side effects of fluoroquinolones, we find tendinitis, tendon rupture, photosensitivity, neurological symptoms, exacerbations of myasthenia gravis, muscle weakness, and QT interval prolongation. The side effects of delafloxacin have also been described, namely, peripheral neuropathy, hypersensitivity, and *Clostridioides* (*Clostridium*) *difficile*-associated diarrhea as possible, but less severe, compared to earlier fluoroquinolones; it is noteworthy to mention that these adverse effects were detected in a dose-dependent manner. The most frequently detected treatment-emergent adverse effects during a delafloxacin therapy were diarrhea, vomiting, and extravasation on the infusion site. Among the uncommon treatment-related complications, we find hyperglycemic episodes (in a single patient out of ten examined) and an elevation of transaminase enzymes (in a single patient out of twenty-two examined). Additionally, a lack of photosensitivity and cardiotoxicity were also demonstrated. According to the side effect profile, delafloxacin is a well-tolerated antibiotic [14,18,23]. Delafloxacin is currently approved for therapy in adults’ acute bacterial skin and skin-structure infections (ABSSSI), as well as for community-acquired bacterial pneumonia (CABP) [23,24,25,26,27]. However, additional potential indications for delafloxacin therapy can be bloodstream infection and intra-abdominal infection [23].

Resistance to fluoroquinolones in Enterobacterales is explained by the accumulation of mutations in gyrase- (*gyrA*, *gyrB*) and topoisomaers IV (*parC*, *parE*)-coding genes. These mutations develop in the specific sequences of *gyrA*, *gyrB, parC*, and *parE* genes, which are called quinolone-resistance-determining regions (QRDRs) [28,29]. Additionally, plasmid-mediated quinolone resistance (PMQR) determinants were described, which enhance the development of fluoroquinolone resistance. PMQR includes Qnr determinants, Aminoglycoside-acetyltransferase-(6′)-Ib-cr variant, and QepA and OqxAB efflux pumps [30,31,32,33].

The aim of this study is the investigation of the antibacterial efficacy of delafloxacin in *Escherichia coli* strains.

## 2. Materials and Methods

### 2.1. Strains

A total of 47 non-repetitive *E. coli* strains were collected between September and December 2022 at South-Pest Central Hospital, National Institute of Hematology and Infectious Diseases, from various clinical samples, including hemoculture and urine. All isolates were identified by matrix-assisted laser desorption ionization time-of-flight mass spectrometry (MALDI Biotyper, Bruker, Bremen, Germany). The inclusion criteria of *E. coli* strains in this study were resistance to ciprofloxacin and/or resistance to third-generation cephalosporins or confirmed extended-spectrum β-lactamase (ESBL) positivity. ESBL positivity was determined by a double-disk synergy test.

### 2.2. Determination of the Minimum Inhibitory Concentration (MIC)

Antibiotic susceptibility testing was performed for delafloxacin, ciprofloxacin, levofloxacin, moxifloxacin, ceftazidime, cefotaxime, and imipenem. MIC values were determined by the broth microdilution method in Muller–Hinton broth in 96-well microplates. The MIC results were interpreted according to the latest EUCAST protocol (www.eucast.org), accessed on 10 January 2023. *E. coli* ATCC 25922 was the control strain.

### 2.3. Whole-Genome Sequencing (WGS)

WGS analysis was performed on two selected *E. coli* strains (ECO-SEOMI-LKH 920/1 and 951/2). The selection criteria were *E. coli* strains exhibiting ciprofloxacin and delafloxacin resistance as well as ESBL phenotype. WGS was performed by the Illumina MiSeq system in Eurofins BIOMI Kft (Gödöllő, Hungary). Briefly, genomic DNA was extracted by the NucleoSpin Microbial DNA Mini kit (Macherey-Nagel, Düren, Germany). The amount of isolated DNA was measured by Qubit fluorometer, and the quality of DNA was tested by microcapillary electrophoresis (Tape Station 4150, Agilent, Waldbronn, Germany). Libraries were prepared by az Illumina DNA Prep kit, according to the manufacturer’s instruction. Sequencing was performed on an Illumina Miseq system using MiSeq Reagent Kit v2 generating 250 bp paired-end reads. Genome assembly was performed with the SPAdes Genome assembler algorithm v3.15.3. Antibiotic-resistance genes were detected in the assembled genomes by Bionumerics v8.1 software.

The assembled genomes of *E. coli* strains (920/1 and 951/2) were submitted to NCBI Genbank at Bioproject PRJNA971108; sequence read archive (SRA) identifiers: SAMN35019574 (ECO-SEOMI-LKH 920/1 strain) and SAMN35019575 (ECO-SEOMI-LKH 951/2 strain).

## 3. Results

**Figure 1:**
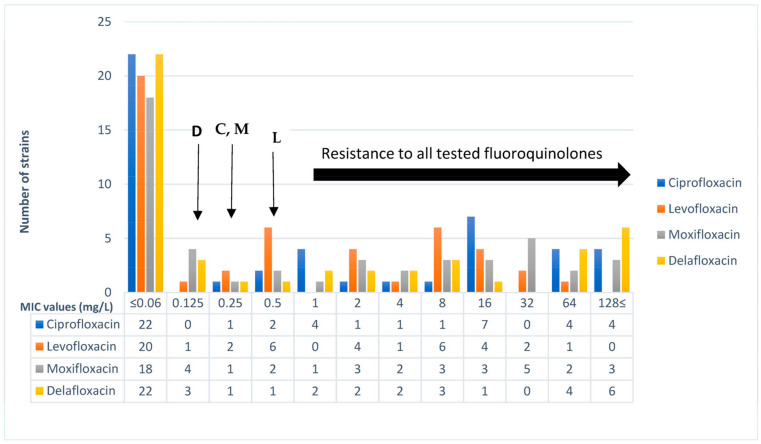
Distribution of fluoroquinolone MIC values of the 47 *E. coli* strains in this study. Arrows indicate EUCAST breakpoint for each fluoroquinolone, namely, D: delafloxacin, C: ciprofloxacin, M: moxifloxacin, and L: levofloxacin.

**Figure 2:**
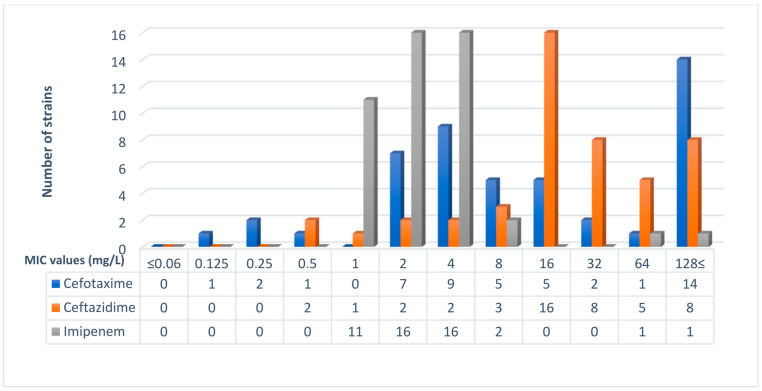
Distribution of the beta-lactam MIC values of the 47 *E. coli* strains in this study. Cefotaxime, ceftazidime, and imipenem MIC values are shown.

The investigated 47 *E. coli* strains represented a wide range of fluoroquinolone MIC distribution. Altogether, 20 of them were susceptible to all tested fluoroquinolones, while on the other hand, 18 were resistant to all tested fluoroquinolones. In our collection, ceftazidime MIC values were in the range of 0.5–128 mg/L and cefotaxime MIC values were in the range of 0.125–128 mg/L. Altogether, 46 *E. coli* strains were confirmed as ESBL producers. Among the investigated strains, 43 out of 47 *E. coli* exhibited imipenem MIC values between 1 and 4 µg/mL (Figure 1 and Figure 2). 

Altogether, 25 out of 47 *E. coli* strains showed sensitivity to delafloxacin, and a single strain was sensitive only to delafloxacin but exhibited resistance to all other fluoroquinolones. The delafloxacin resistance rate was 47% (22/47), ciprofloxacin resistance was 51% (24/47), moxifloxacin resistance was 51% (24/47), and levofloxacin resistance was 38% (18/47). MIC50 and MIC90 values were determined in our study. These values indicate the MIC value of 50% and 90% of the tested strains, respectively. In our collection, the MIC50 value was 0.125 mg/L for delafloxacin and all other fluoroquinolones had 0.25 mg/L. The MIC90 values for delafloxacin, ciprofloxacin, moxifloxacin, and levofloxacin were 64 mg/L, 64 mg/L, 32 mg/L, and 16 mg/L, respectively. In our collection, 20 *E. coli* strains were delafloxacin-susceptible but exhibited an ESBL phenotype and ciprofloxacin resistance. The *E. coli* strains exhibiting ciprofloxacin MIC value above 1 mg/L were resistant to delafloxacin. The *E. coli* strains exhibiting 4 and 8 mg/L delafloxacin MIC values were moxifloxacin-resistant.

**Figure 3:**
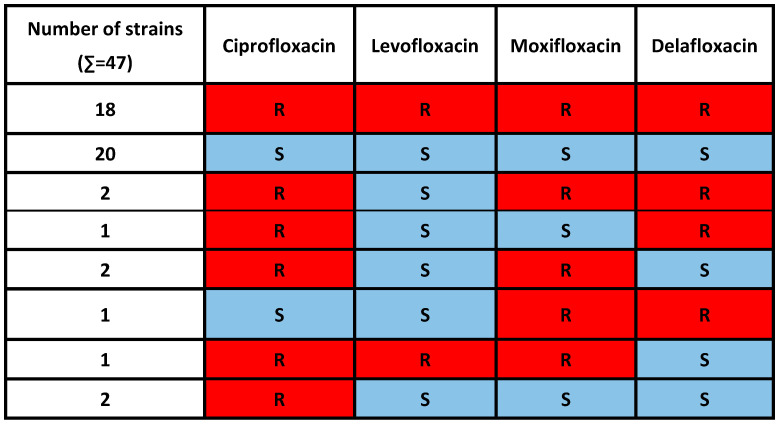
Fluoroquinolone resistance patterns of the 47 *E. coli* strains. R: resistant, S: susceptible.

In our study, unusual phenotypes were also detected. The levofloxacin MIC values did not strongly correlate with other fluoroquinolones. Two *E. coli* strains were levofloxacin-susceptible but resistant to all other tested fluoroquinolones. Two *E. coli* strains were susceptible to levofloxacin and delafloxacin, but resistant to other fluoroquinolones. A single *E. coli* strain was levofloxacin- and moxifloxacin-susceptible, but resistant to other fluoroquinolones. Another strain was ciprofloxacin- and levofloxacin-susceptible but resistant to other fluoroquinolones. Furthermore, two strains were resistant only to ciprofloxacin, but were susceptible to all other fluoroquinolones. The data are summarized in Figure 3.

**Figure 4:**
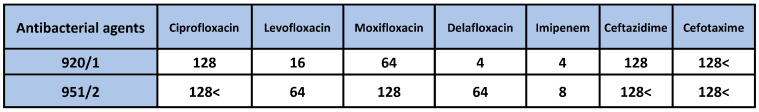
MIC values of the two *E. coli* strains selected for whole-genome sequencing.

**Figure 5:**
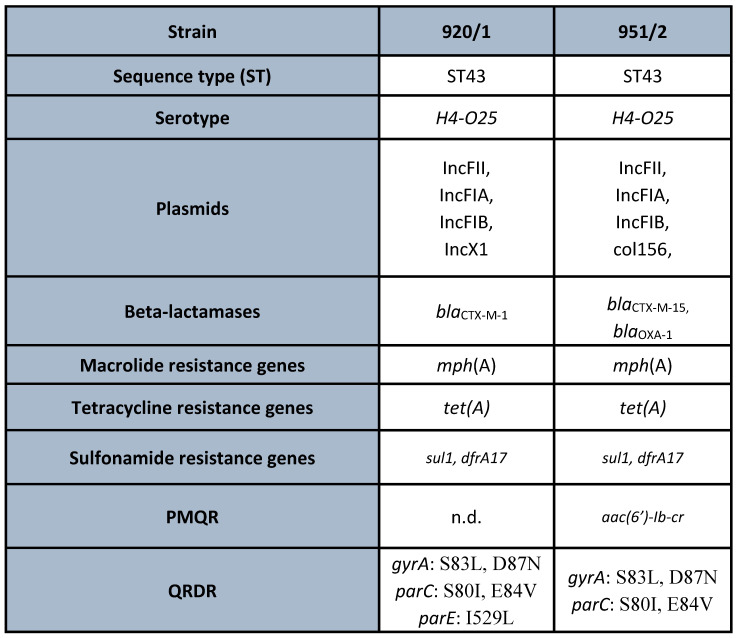
Results of the whole-genome sequencing of the two *E. coli* strains (920/1 and 951/2). PMQR: plasmid-mediated quinolone resistance, QRDR: quinolone-resistance-determining region, n.d.: not detected.

Based on the results of the susceptibility testing, two multidrug-resistant (MDR) strains, namely, ECO-SEOMI-LKH 920/1 and 951/2, were selected for WGS analysis. The MIC values are presented in Figure 4. Both strains belong to *E. coli* ST43 international high-risk clone and both carried diverse resistance genes, including ESBLs, namely, *bla*_CTX-M-1_, *bla*_CTX-M-15_. In the case of *E. coli* 920/1, five amino acid alterations in multiple positions of QRDR, namely, *gyrA* S83L, D87N, *parC* S80I, E84V, and *parE* I529L, were detected. *E. coli* 951/2 was marked by similar multiple mutations of QRDR; namely, *gyrA* S83L, D87N and *parC* S80I, and E84V were identified. Among the PMQR determinants, the bifunctional Aminoglycoside-acetyltransferase(6′)-Ib-cr was detected in this strain. Further resistance mechanisms were also detected, which are summarized in Figure 5.

## 4. Discussion

In our study, we investigated the delafloxacin-resistance rate in 47 *E. coli* strains that were isolated from clinical samples (e.g., hemoculture and urine). Delafloxacin is a novel fluoroquinolone agent, which was approved for clinical application in recent years. Our results demonstrate that 47% (22/47) of the *E. coli* strains exhibited resistance to delafloxacin compared to ciprofloxacin resistance (51% (24/47)), moxifloxacin resistance (51% (24/47)), and levofloxacin resistance (38% (18/47)). The MIC50 value of delafloxacin was 0.125 mg/L, while on the other hand, all other fluoroquinolone agents had a 0.25 mg/L MIC50 value. Despite of the fact that delafloxacin is not currently available in our country for clinical use, it can be clearly seen that *E. coli* can be selected from its resistant subtypes against this new fluoroquinolone agent. Furthermore, we also detected that ciprofloxacin and moxifloxacin resistance are frequently associated (20/47) to delafloxacin resistance. Notably, *E. coli* strains exhibiting ciprofloxacin MIC above 1 mg/L were already resistant to delafloxacin. This phenotype can be used as a marker to indicate delafloxacin resistance.

Our results also show indications for the clinical application of delafloxacin, as 20 *E. coli* strains were delafloxacin-susceptible but exhibited an ESBL phenotype and ciprofloxacin resistance. On the other hand, we also found 22 delafloxacin-resistant *E. coli* strains that exhibited an ESBL phenotype.

Interestingly, levofloxacin MIC values did not strongly correlate to other fluoroquinolones in this study. We detected two *E. coli* strains with delafloxacin resistance, but one strain was sensitive to ciprofloxacin and levofloxacin, while the other was sensitive to levofloxacin and moxifloxacin (Figure 3).

In our study, we selected two MDR *E. coli* strains for WGS analysis. Both strains belong to the ST43 international high-risk clone of *E. coli* based on Pasteur’s multilocus sequence typing (MLST) database. Interestingly, the *E. coli* ST43 in Pasteur’s MLST database corresponds to ST131 in the Achtman MLST scheme [34,35,36]. The high-risk clones of *E. coli* are disseminated worldwide, and these carry diverse resistance mechanisms, including ESBLs (e.g., *bla*_CTX-M_, *bla*_SHV_, *bla*_TEM_), carbapenemases (e.g., *bla*_KPC_, *bla*_NDM_, *bla*_VIM_, *bla*_IMP_, *bla*_OXA-48_), and colistin-resistance determinants (e.g., *mcr*). Furthermore, these high-risk clones are causative agents of a high number of hospital-acquired infections [37,38].

The predominant *E. coli* high-risk clone is ST131; however, additional high-risk clones are also known, namely, ST10, ST69, ST73, ST405, ST410, ST457, and ST1193 [37,38,39]. 

*E. coli* ST131 is a multi-resistant high-risk clone and it is responsible for the spreading of resistance determinants. The two major serotypes of *E. coli* ST131 are O16:H5 and O25:H4. Additionally, *E. coli* ST131 has three *fimH* alleles, namely, 41, 22, and 30. According to antibiotic-resistance patterns, ST131 is also classified into subclones H30R and H30Rx [37,38]. Originally, the *E. coli* ST131 clone was reported as O25b:H4 serotype, and CTX-M-type ESBL production was usually detected. The majority of strains in the ST131 clone are usually resistant to several antibiotics, including cephalosporins, carbapenems, aminoglycosides, fluoroquinolones, sulfonamides, nitrofurantoin, and tetracycline [37].

The ST131 clone is the main member of the phylogroup B2 of *E. coli,* according to the phylogenetic analysis of whole-genome data. *E. coli* ST131 is also recognized as the origin of different sequence types, namely, ST1680, ST1982, ST1461, and ST1193. 

High number and diverse virulence factors are usually detected in *E. coli* strains belonging to phylogroup B2 [37]. Unlike other B2 *E. coli* strains, ESBL production and fluoroquinolone resistance are commonly detected in ST131. The phylogeny of ST131 has been clustered into three major clades based on resistance traits and population genetics. The three clades are A, B, and C. Among the clades, the A/H41 clade and B/H22 clade are smaller subgroups. However, ST131 strains in clade A have been reported in many community-acquired infections worldwide, and these strains have also been reported in stool samples of healthy children in China [37,38]. Clade A *E. coli* ST131 has been reported in environmental samples, namely, in water from the Jurong river reservoir in Singapore. Interestingly, the strains of clade B have been described to colonize poultry, contaminate meat, and carry colistin-resistance genes (e.g., *mcr-1* and *mcr-3*); therefore, these strains are considered as foodborne pathogens. The strains of clade B have been isolated from several human infections, notably, from clinical samples of urine, blood, and peritoneal fluid. However, at present, the most challenging clade is related to the strains of clade C. This clade originates from clade B, and it can be divided into two major subclades, namely, C1/H30-R and C2/H30-Rx. *E. coli* strains of clade C usually carry *bla*_TEM_; additionally, subclade C1 usually carry *bla*_CTX-M-14_ or *bla*_CTX-M-27_ ESBL genes, and by contrast, subclade C2 is mainly associated with *bla*_CTX-M-15_ [37,38].

To date, *E. coli* ST43 was described from clinical isolates in some countries. In Panama, Central America, it was detected as *bla*_CTX-M-15_-positive and ciprofloxacin-resistant [34]. In Italy, *E. coli* ST43 was detected as KPC-3-positive and fluoroquinolone-resistant [40]. *E. coli* ST43 in USA was reported as fluoroquinolone-resistant having multiple QRDR mutations, but it was susceptible to third-generation cefalosporins and carbapenems [41].

In our study, the two strains of *E. coli* ST43 were ESBL-positive, harboring *bla*_CTX-M-1_ and *bla*_CTX-M-15_, and both strains were resistant to all tested fluoroquinolone agents, including delafloxacin (Figure 5). The genetic background of fluoroquinolone resistance in these two strains of *E. coli* ST43 demonstrated the accumulation of multiple QRDR mutations. In the case of *E. coli* 920/1, five mutations were present: *gyrA* S83L, D87N, *parC* S80I, E84V, and *parE* I529L, while in *E. coli* 951/2, four mutations, *gyrA* S83L, D87N and *parC* S80I, E84V plus *aac(6′)-Ib-cr* PMQR determinant, were detected (Figure 5).

Fluoroquinolone resistance in Enterobacterales is frequently detected, especially in MDR international high-risk clones, such as in *E. coli* ST131, ST1193, ST69, and CC10 strains [38,42,43,44], as well as in *K. pneumoniae* ST11, ST15, ST101, ST147, and ST307 [45,46,47,48,49,50].

It has been demonstrated that diverse fitness cost is associated with fluoroquinolone resistance in different clones of *K. pneumoniae* and *E. coli*. High-risk clones of *K. pneumoniae* and *E. coli* suffer a lower fitness cost and retain a favorable fitness after the development of fluoroquinolone resistance, which enable them to survive and disseminate [51,52]. These features are usually seen in high-risk clones because they can persist and spread for a longer period of time in hospital environments and cause different infections. Additionally, high-risk clones can acquire further resistance determinants during dissemination in hospital settings [37,47]. Double serin mutations in the QRDR sequences of the *gyrA* and *parC* genes are linked to high-risk clones, which are associated with a favorable fitness [53]. Our results correlate well to these earlier data because we detected fluoroquinolone resistance in the *E. coli* ST43 international high-risk clone, which is associated with double serin mutations in the QRDR sequences of the *gyrA* and *parC* genes.

To date, delafloxacin resistance has been scarcely reported among bacterial isolates. According to the available data, delafloxacin resistance was reported in *S. aureus*, *Neisseria gonorrhoeae*, and non-tuberculous Mycobacteria [54,55,56]. 

In conclusion, in our study, we analyzed the efficacy of delafloxacin in a collection of 47 *E. coli* strains. A limitation of this study is that we investigated only 47 strains; however, these early results are useful to gain insights into the possible clinical use of delafloxacin. We detected a remarkably high (47%) delafloxacin resistance among MDR *E. coli* strains. This report can be considered as a baseline result, because delafloxacin is not yet available for clinical application in Hungary. We also reported the detection of the *E. coli* ST43 high-risk international clone in Hungary. This clone has been reported in several countries with different resistance determinants, and in our study, both strains of *E. coli* ST43 exhibited ciprofloxacin and delafloxacin resistance together with CTX-M-1- and CTX-M-15-type ESBL production.

## Data Availability

The assembled genomes of *E. coli* strains (920/1 and 951/2) were submitted to NCBI Genbank at Bioproject PRJNA971108; sequence read archive (SRA) identifiers: SAMN35019574 (ECO-SEOMI-LKH 920/1 strain) and SAMN35019575 (ECO-SEOMI-LKH 951/2 strain).
